# A Rare Case of Pediatric Pulmonary Sarcoidosis Without Lymph Node Involvement Presenting With Centrilobular Ground-Glass Opacities

**DOI:** 10.7759/cureus.91938

**Published:** 2025-09-09

**Authors:** Diane Choi, Julia Small, Denzil Reid, Carolyn Garcia, Katherine Harer

**Affiliations:** 1 Department of Pediatrics, UMass Chan Medical School - Baystate, Springfield, USA; 2 Department of Pediatrics, UMass Chan Medical School, Worcester, USA; 3 Department of Medicine, UMass Chan Medical School - Baystate, Springfield, USA

**Keywords:** cryptogenic organizing pneumonia, diagnostic error, ground glass opacities, high-value care, pediatric hospital medicine, pulmonary sarcoidosis, sarcoidosis

## Abstract

A 17-year-old previously healthy male presented with acute hypoxic respiratory failure after one month of progressively worsening dyspnea on exertion, non-productive cough, fevers, and weight loss. He was initially discharged on oral steroids with a working diagnosis of cryptogenic organizing pneumonia, but was readmitted after one month for acute hypoxic respiratory failure. Extensive laboratory workup, including serum angiotensin converting enzyme level, hypersensitivity pneumonitis panel, and bronchoalveolar lavage fluid cultures, was normal. Computed tomography scan of the chest revealed diffuse centrilobular ground-glass opacities and a 4-mm solid right lower lobe pulmonary nodule, and he underwent a video-assisted thoracoscopic surgery (VATS) procedure with segmental resection of the lingula. Pathology ultimately showed non-necrotizing granulomatous inflammation in the interstitium of the lung parenchyma, airways, and blood vessels, consistent with sarcoidosis. Diagnosis of interstitial lung disease is difficult when histologic diagnosis requires an invasive, costly VATS biopsy, but prompt recognition of sarcoidosis is critical to prevent potentially fatal pulmonary fibrosis or cardiac sarcoidosis. To our knowledge, this is the first case report of pediatric pulmonary sarcoidosis with ground-glass opacities lacking any lymphadenopathy, skin, or neurologic manifestations.

## Introduction

Both cryptogenic organizing pneumonia and sarcoidosis are considered diffuse parenchymal lung diseases, a “heterogeneous group of nonneoplastic disorders resulting from damage to the lung parenchyma by varying patterns of inflammation and fibrosis” [1]. This broad definition is characterized clinically by respiratory symptoms and a variety of bilateral radiographic features, and as such, encompasses a huge number of distinctly different pathophysiologies. Identifying the correct diagnosis, therefore, is a dynamic process between clinician, radiologist, and pathologist, and the idea of a “gold standard” histological diagnosis has largely been replaced by this multidisciplinary approach [2]. This is particularly when the highest-yield pathology is generally found in video-assisted thoracoscopic surgery (VATS) with wedge biopsies, which are invasive, inconvenient, and costly [3].

We report a case of pediatric sarcoidosis that was initially identified as cryptogenic organizing pneumonia in a conservative approach to diagnosis, but ultimately required the VATS wedge biopsy to appropriately recognize the right etiology, which was critical in his successful treatment, resolution of symptoms, and thorough evaluation for extrathoracic manifestations. Sarcoidosis has widespread variation in radiographic and pathologic presentations, but, to our knowledge, this is the first pediatric case with radiographic evidence of ground-glass opacities, a non-specific finding of interstitial lung disease, but a histologic finding of granulomas, small clusters of immune cells consistent with a diagnosis of sarcoidosis.

This case was previously presented as a poster at the 2024 Pediatric Hospital Medicine Conference on August 3, 2024, and the 2024 CHEST Annual Meeting on October 9, 2024.

## Case presentation

A 17-year-old previously healthy male presented with acute hypoxic respiratory failure after one month of progressively worsening dyspnea on exertion, non-productive cough, intermittent low-grade fevers, and 4.4 kg weight loss. He initially presented to an urgent care center, where he was diagnosed with acute bronchitis with bronchospasm and treated with five days of 40 mg prednisone and albuterol. He did not respond to treatment and returned on three additional occasions for persistent symptoms. History was notable for pet exposure (dogs, cats, turtles, and a bearded dragon), but no recent travel, sick contacts, or exposure to construction, inhalants, or second-hand smoke. He denied recreational substance use, including cannabis and vaping. He was a non-Hispanic white male with no family history of autoimmune or respiratory diseases.

**Figure 1:**
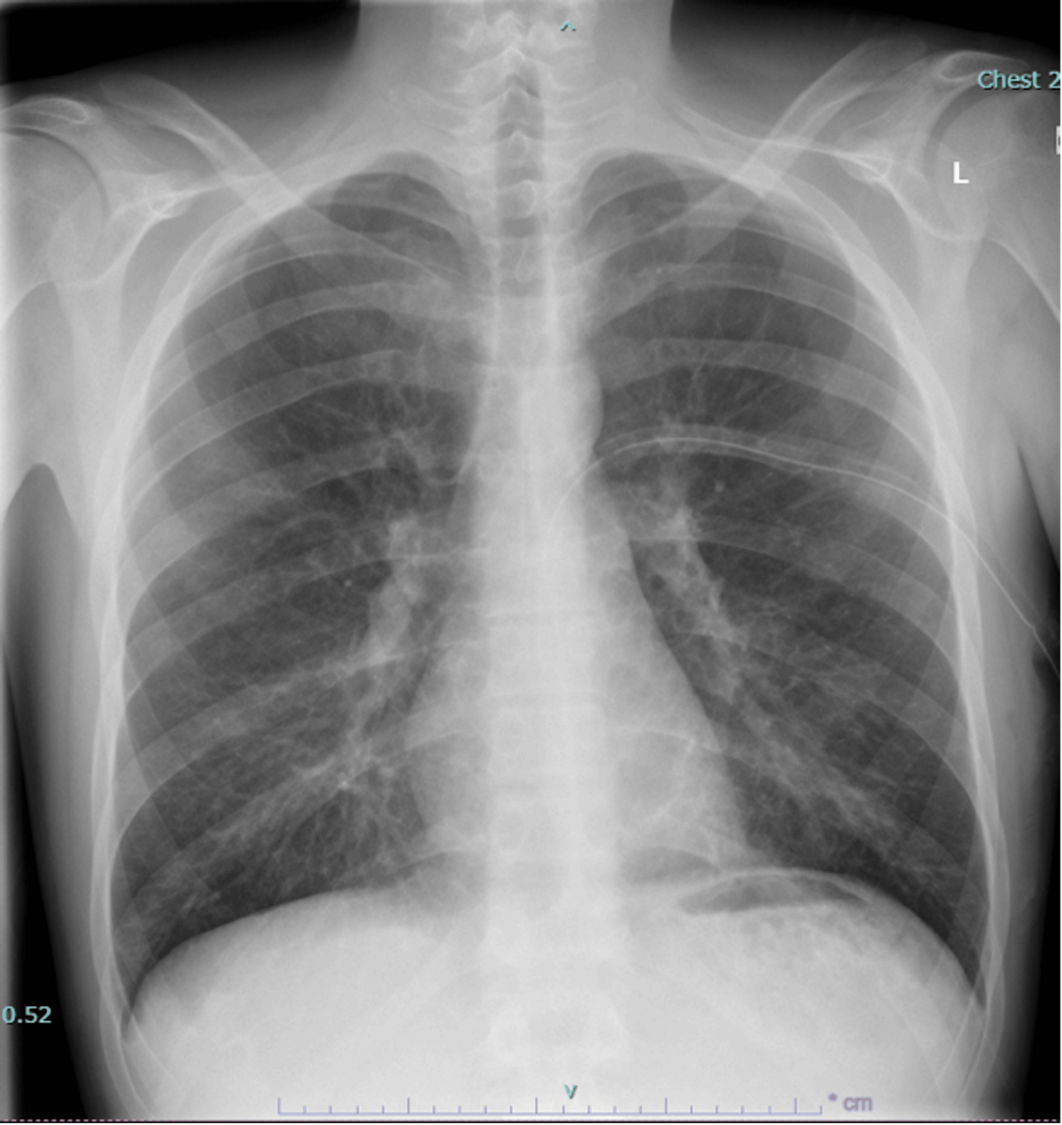
Normal chest X-ray with no acute cardiopulmonary process.

Differential diagnosis at the time included upper respiratory illness vs. myopericarditis. Outpatient workup performed was within normal limits, including a complete blood count with differential, comprehensive metabolic panel, including calcium, D-dimer, inflammatory markers, thyroid function testing, Lyme serologies, Northeast tick panel, blood cultures, mononucleosis antibody test, HIV serologies, N-terminal pro-brain natriuretic peptide, and troponin (Table 1). Chest X-ray was negative for cardiopulmonary disease (Figure 1). EKG showed a normal sinus rhythm. A transthoracic echocardiogram was obtained, which showed no significant abnormalities.

**Table 1:** Serology laboratory findings during hospitalization were largely normal, with the exception of a positive ANA with titers 1:80 in a speckled pattern. WBC, white blood cell count; BUN, blood urea nitrogen; AST, aspartate aminotransferase; ALT, alanine aminotransferase; CRP, C-reactive protein; ESR, erythrocyte sedimentation rate; Nt-Probnp, B type natriuretic peptide; HSTnT, high sensitivity troponin T; TSH, thyroid-stimulating hormone; ACE, angiotensin converting enzyme; ANA, anti-nuclear antibody; PCR, polymerase chain reaction.

Component	Reference range	Results
WBC	4.0-11.0 k/mm3	7.7
Neutrophils	44-76%	64.2
Lymphocytes	15-43%	26.7
Eosinophils	0-6%	0.9
Monocytes	4.5-10.5%	7.4
Basophils	0-2%	0.5
Hemoglobin	13.0-16.0 Gm/dL	16.8
Platelets	150-460 k/mm3	271
Sodium	1330145 mmol/L	133
Potassium	3.6-5.2 mmol/L	4.7
Chloride	988-107 mmol/L	96
Bicarbonate	22-29 mmol/L	26
BUN	5-18 mg/dL	15
Creatinine	0.7-1.2 mg/dL	0.8
Glucose	70-99 mg/dL	98
AST	0-40 units/L	24
ALT	0-41 units/L	19
CRP	<0.3 mg/dL	<0.3
ESR	0-15 mm/hr	6
D-dimer	<1.01 mg/L FEU	0.25
Nt-Probnp	0-125 pg/mL	19
HSTnT	<22 ng/L	9
TSH	0.68-4.09 uIU/mL	2.24
ACE	14-82 U/L	81
ANA	Negative	Positive, speckled pattern, 1:80
Cryptococcal antigen	Negative	Negative
Infectious mononucleosis antibody test	Negative	Negative
Legionella pneumophila antigen	Negative	Negative
Lyme disease antibody screen	Negative	Negative
Blastomyces antibody	Negative	Negative
HIV 4^th^-generation antigen-antibody result	Negative	Negative
Anaplasma PCR	Not detected	Not detected
Babesia microti PCR	Not detected	Not detected
Fungitell (beta D glucan)	<80 pg/mL	40
Coccidioides antibody	Negative	Negative
QuantiFERON 1 tube result	Negative	Negative

**Figure 2:**
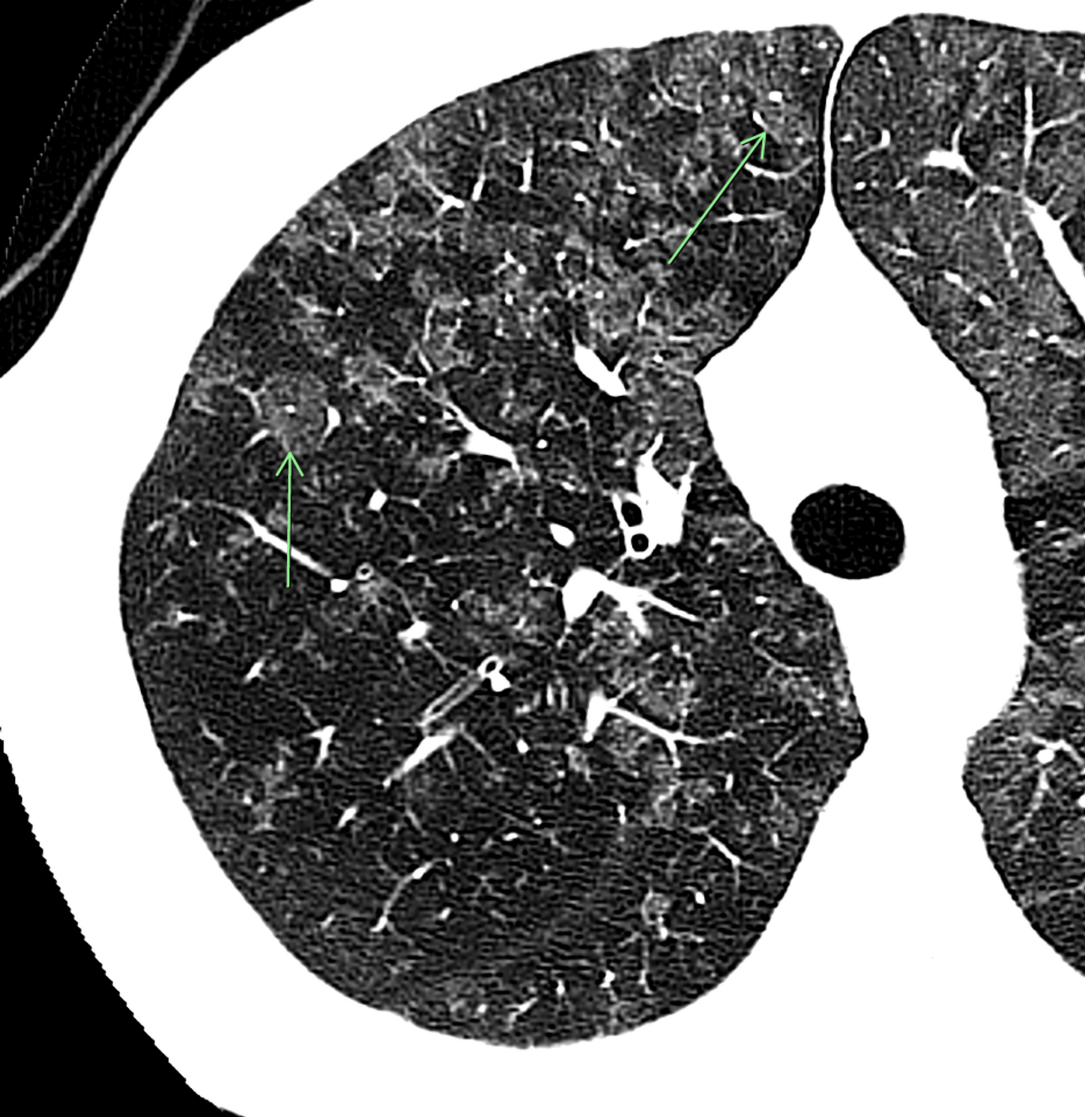
Magnified view of the right lung shows diffuse, scattered centrilobular ground-glass opacities. The same pattern was seen in both lungs. The arrows indicate centrilobular ground-glass opacities.

Upon arrival, he was afebrile, tachypneic to 39 breaths per minute, tachycardic to 139 beats per minute, and hypoxic requiring bilevel positive airway pressure (BiPAP) respiratory support. Additional laboratory studies included a negative anti-nuclear antibody (ANA) screen (1:80), serum angiotensin converting enzyme (ACE), expanded respiratory viral panel, blastomycosis, coccidioidomycosis, legionella, and QuantiFERON gold (Table 2). Computed tomography (CT) scan of the chest revealed diffuse primarily centrilobular ground-glass opacities and a 4-mm solid right lower lobe pulmonary nodule (Figure 2).

**Table 2:** Bronchoalveolar lavage results demonstrated minimal neutrophilia, with lymphopenia and a decreased CD4/CD8 ratio, and were negative for infectious organisms on PCR. WBC, white blood cell count; RBC, red blood cell count; PCR, polymerase chain reaction.

Component	Reference range	Results
Bacterial 16s broad-range PCR	No bacterial DNA detected	No bacterial DNA detected
Fungal broad-range PCR	No fungal DNA detected	No fungal DNA detected
Nontuberculous mycobacteria PCR	No nontuberculous Mycobacteria	No nontuberculous Mycobacteria
Mycobacterium tuberculosis complex PCR	No Mycobacterium tuberculosis complex DNA	No Mycobacterium tuberculosis complex DNA
Pneumocystis smear result	Negative	Negative
Color	Colorless	Colorless
WBC, fluid	Not well established	233 per mm^3^
RBC, fluid	Not well established	109 per mm^3^
Segmented neutrophils, fluid	Not well established	8%
Lymphocytes, fluid		13%
Eosinophils, fluid		4%
Macrophages, fluid		75%
Cd4/Cd8 ratio	1.100-1.400	0.91

He was started on high-dose steroids of 60 mg daily and *Pneumocystis jirovecii* prophylaxis. A bronchoalveolar lavage (BAL) was done, and the fluid was negative for bacteria, fungus, and acid-fast bacilli. The BAL fluid showed minimal neutrophilia (8%) and lymphopenia (13%), not enough to suggest an infectious process, particularly with negative acid-fast bacilli stains, fungal, and bacterial stains, as well as a decreased CD4/CD8 ratio of 0.91 (Table 2). The single 4-mm node on chest CT was determined to be too small to capture on needle biopsy. VATS with wedge biopsy was discussed with the family, but deferred at the time due to rapid clinical improvement and stability on oral steroids. He was discharged home with a working diagnosis of cryptogenic organizing pneumonia with a six-month steroid taper and close follow-up. He tolerated the treatment well and was weaned to 40 mg/day of prednisone after 4.5 weeks, but that week developed worsening shortness of breath and returned to the hospital. Given the inability to wean from high-dose steroids, he underwent a VATS procedure with segmental resection of the lingula. Pathology showed non-necrotizing granulomatous inflammation in the interstitium of the lung parenchyma, airways, and blood vessels, consistent with sarcoidosis.

## Discussion

The prompt recognition of sarcoidosis is critical to provide appropriate therapeutic interventions and prevent potentially fatal pulmonary fibrosis or cardiac sarcoidosis, but numerous diagnostic challenges impeded our ability to correctly diagnose this patient [3,4]. Clinically, our patient presented with the classic pulmonary symptoms of sarcoidosis, such as non-productive cough and dyspnea, but lacked the pathognomonic hilar lymphadenopathy or any extrapulmonary manifestations. The rare case reports of patients with ground-glass opacities on chest CT due to sarcoidosis also underwent extensive work-up prior to diagnosis, but to our knowledge, this is the first case report of such a presentation lacking any lymphadenopathy, skin, and/or neurologic manifestations [4,5]. This pediatric presentation is additionally unique in a 17-year-old male, as juvenile disease, while rare, usually presents from ages 13 to 15 [6].

In an effort to avoid invasive diagnostic testing, a number of additional hematologic studies were performed, which, while able to exclude many alternative etiologies such as hypersensitivity pneumonitis and fungal or mycobacterial infections, were unable to detect sarcoidosis. In particular, while serum ACE levels are a promising biomarker of disease activity and granuloma burden, they demonstrate only a 60% sensitivity in the diagnosis of sarcoidosis with high variability and controversy [7]. Moreover, several studies have shown that activity was greater in children compared to adults [8].

The lack of lymph node involvement on CT imaging suggests this patient has stage III thoracic sarcoidosis by the Scadding classification, a presentation found in only 10-12% of patients at diagnosis [9-11]. Even the parenchymal involvement in this case is atypical, lacking characteristic peri-lymphatic micronodules or ground-glass opacities localized to the upper and middle lobes [3,4,11].

Although high-resolution CT has been shown to have more utility than other diagnostic modalities, especially in staging of the disease, BAL is also a useful supplement to the diagnosis of thoracic sarcoidosis [12,13]. In sarcoidosis, CD4+ T lymphocyte cells form non-caseating granulomas, which should show lymphocytosis and elevated CD4/CD8 ratios (>2-4), were not found in our case [13]. However, even this proves to have a low sensitivity of 0.70 and is not sufficient for the diagnosis of sarcoidosis, even with supportive evidence. Ultimately, despite our best efforts, VATS with wedge biopsy was the only way forward.

An accurate diagnosis allowed for a thorough evaluation for extrathoracic complications, such as cardiac involvement or uveitis, which was negative, and to plan an appropriate treatment regimen. However, disease severity and prognosis are highly variable, which makes management challenging [3]. For example, our patient experienced a sudden worsening of his symptoms after weaning to 40 mg of prednisone, a standard starting dose for control of symptoms. Fortunately, the patient tolerated the steroids without side effects and was able to be weaned off before requiring glucocorticoid-sparing agents such as methotrexate or azathioprine.

## Conclusions

This case highlights a rare and atypical presentation of pulmonary sarcoidosis that led to a delay in diagnosis. The patient had the characteristic pulmonary symptoms; however, workup showed normal calcium, normal ACE level, no lymphadenopathy, atypical imaging, and nonsignificant results on BAL, making other differential diagnoses more likely. As sarcoidosis is a systemic disorder and can present with a wide variety of symptoms and signs, it is important to consider it as a diagnosis, especially in patients with subacute respiratory symptoms without a clear etiology, regardless of age.

In the pursuit of high-value care and shared decision-making, the patient was discharged with a presumptive diagnosis that was ultimately proven incorrect on subsequent readmission for invasive testing. It is our hope that by modeling this approach and describing a rare presentation of pediatric sarcoidosis presenting as a diffuse parenchymal lung disease, we equip other providers to recognize the subtle features that resulted in delayed diagnosis for our patient.
